# Physical, Rheological and Microstructural Properties of Asphalt Modified by Low-Molecular-Weight Polyolefin

**DOI:** 10.3390/ma19030571

**Published:** 2026-02-02

**Authors:** Jun He, Binbin Leng, Meizhu Chen, Shijie Guo, Jingjun Yu

**Affiliations:** 1 State Key Laboratory of Silicate Materials for Architectures, Wuhan University of Technology, Wuhan 430070, China; hejun0607@whut.edu.cn (J.H.); 331450@whut.edu.cn (J.Y.); 2 Qingdao Guantong Municipal Construction Co., Ltd., Qingdao 266000, China; my200678@163.com; 3 School of Materials Science and Engineering, Wuhan University of Technology, Wuhan 430070, China

**Keywords:** asphalt, polyolefin modifier, rheological properties, microstructure, physical properties

## Abstract

Improving both the high- and low-temperature performance of asphalt is still difficult in modern pavement applications. This performance imbalance has motivated the development of new modification strategies that can enhance temperature stability while maintaining construction workability. In this research, a low-molecular-weight elastic polyolefin (POL) with inherent compatibility was introduced as a novel asphalt modifier. POL was incorporated at five dosages (0%, 2%, 4%, 6%, and 8% by weight of asphalt) to investigate its effects on the fundamental physical, rheological, and low-temperature properties of the asphalt. The rheological behavior was characterized by dynamic shear rheometer (DSR) and bending beam rheometer (BBR), while the modification mechanism and dispersion morphology were analyzed through Fourier-transform infrared spectroscopy (FT-IR) and fluorescence microscopy (FM). The results reveal that POL markedly improves the high-temperature performance and workability of asphalt, with the rutting factor increasing by two- to eightfold. POL modification improved the thermal stability of asphalt, shifting the maximum decomposition temperature from 455.2 °C for the base binder to 461–463 °C, while the total mass loss remained nearly constant at 80–83%. Microscopic observations confirm that POL forms a physically blended network within the asphalt matrix, exhibiting a green fluorescent structure that becomes progressively continuous with increasing dosage. The most homogeneous dispersion and optimal compatibility occur at a POL dosage of 6%, beyond which phase segregation emerges and low-temperature properties deteriorate. Accordingly, a 6% POL dosage is recommended for achieving balanced performance. These findings provide theoretical and practical guidance for the development of balanced performance and thermally stable POL-modified asphalt materials.

## 1. Introduction

Asphalt pavement is a fundamental component of modern transportation infrastructure, directly influencing road safety, service quality, and the sustainability of traffic networks. With the continuous expansion of highway systems and the increasing frequency of extreme weather events, conventional asphalt materials are facing severe challenges [1,2,3]. Distresses such as rutting, moisture damage, and thermal cracking have become more prevalent, leading to premature pavement deterioration and increased maintenance costs [4,5]. Moreover, oxidative aging progressively reduces the flexibility and adhesion of asphalt binders, further aggravating these issues. To achieve durable and sustainable pavements, it is essential to develop modification strategies that can effectively improve the high-temperature stability, moisture resistance, and aging durability of asphalt binders [6,7]. Therefore, developing effective modification strategies to enhance high-temperature stability, moisture resistance, and aging durability of asphalt binders remains a critical research focus.

Polymer modification has been widely recognized as one of the most effective methods to enhance the performance of asphalt binders. Among various modifiers, the styrene–butadiene–styrene (SBS) copolymer has been most widely used due to its ability to significantly improve elasticity and temperature sensitivity [8,9,10], crumb rubber modification enhances low-temperature ductility and moisture resistance, and thermosetting resins such as epoxy and polyurethane provide excellent stiffness and aging resistance [11]. Despite their outstanding performance, these materials are often associated with high production costs, complex processing, and poor recyclability, limiting their potential for large-scale and sustainable application [12,13].

Thermoplastic polyolefins such as polyethylene (PE) and polypropylene (PP) have recently attracted considerable attention as alternative asphalt modifiers owing to their low cost, wide availability, and environmental advantages [14,15,16,17]. However, the inherently nonpolar molecular structure of conventional polyolefins results in poor compatibility with asphalt binders, leading to phase separation and unstable modification performance [18]. Various strategies, such as chemical functionalization, blending with compatibilizers, and molecular structure regulation, have been proposed to address these limitations, but they often introduce additional processing complexity or cost.

To address these limitations, a low-molecular-weight elastic polyolefin copolymer synthesized from butadiene, ethylene, and styrene has been developed [19]. This copolymer combines the flexibility of elastomers with the thermoplastic characteristics of polyolefins and incorporates a limited number of polar functional groups, thereby improving its compatibility with the asphalt matrix. Previous studies have reported that this material can effectively enhance rutting resistance, improve moisture stability, and reduce mixing and compaction temperatures, indicating promising engineering applicability [20,21,22]. However, the modification mechanism, dosage-dependent performance evolution, and microstructural characteristics of this polyolefin-modified asphalt have not yet been fully elucidated. Considering the critical role of binder properties in governing asphalt mixture performance, a systematic investigation into the physical, rheological, storage stability, and microstructural behavior of this elastic polyolefin-modified asphalt is necessary to support its rational design and practical application.

**Figure 1:**
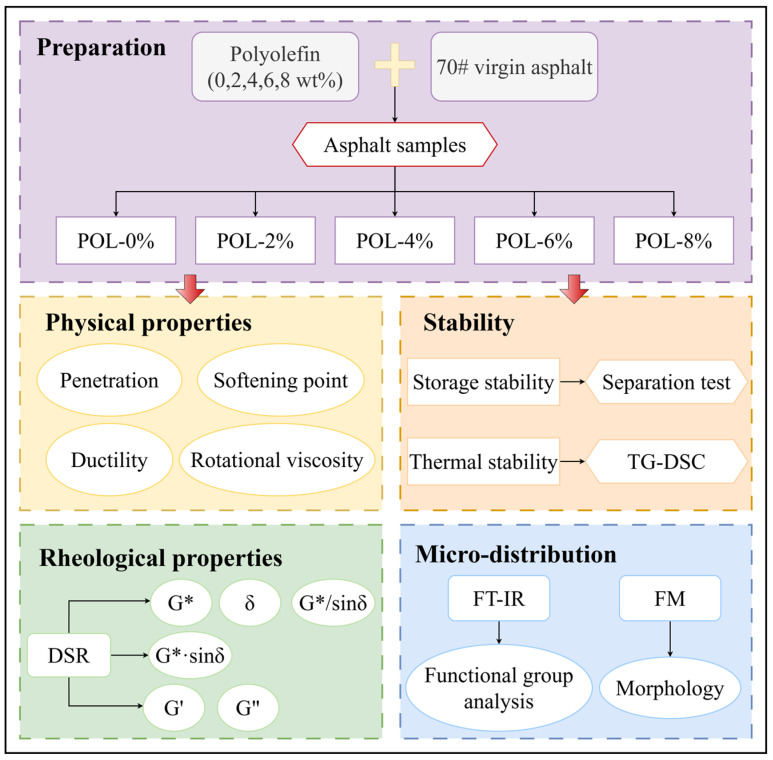
Technology roadmap of this research.

Therefore, this research aims to comprehensively investigate the effects and mechanisms of polyolefin modification on the physicochemical and rheological behavior of base asphalt. The fundamental physical properties were characterized through penetration, softening point, ductility, and viscosity tests. Thermal stability was evaluated using thermogravimetric analysis and differential scanning calorimetry. The viscoelastic response under different temperatures and loading conditions was analyzed by a dynamic shear rheometer, while Fourier-transform infrared spectroscopy and fluorescence microscopy were used to examine the evolution of chemical structures and micro-morphology. By correlating macroscopic performance with microscopic features, this research elucidates the interaction mechanisms between polyolefin and asphalt binders, providing theoretical insights for optimizing polyolefin-based modifiers; the research process is illustrated in Figure 1. The findings are expected to offer new perspectives for developing durable, cost-effective, and environmentally sustainable asphalt pavement materials.

## 2. Materials and Methods

### 2.1. Materials

The virgin asphalt used in this research was 70# matrix asphalt, and its physical properties were determined in accordance with the Chinese standard test method JTG 3410-2025 [23]. The fundamental physical parameters of the asphalt are presented in Table 1. Meanwhile, the polyolefin (POL) employed as the modifier was a special additive product supplied by Honeywell (Shanghai, China) Co., Ltd., and its main performance indicators are summarized in Table 2. The POL used in this study was a commercially available industrial product with mature large-scale production capability, and its application in asphalt modification requires no additional synthesis process.

### 2.2. Sample Preparation

Asphalt samples were prepared through a melt blending technique. Specifically, POL was introduced at levels corresponding to 2%, 4%, 6%, and 8% by weight of asphalt, with each dosage separately mixed into the preheated asphalt matrix. To ensure experimental consistency and comparability, the same mixing duration was applied for all POL dosages. Preliminary trials confirmed that the selected mixing time was sufficient to achieve homogeneous dispersion. The stirring process was conducted for 10 min to ensure thorough blending. Then asphalt samples were shear mixed at a high speed of 4000 r/min for 30 min at 160 °C [6]. Subsequently, the samples were allowed to sit at 160 °C for an additional 20 min. The process parameters detailed above were determined through initial experimental investigations and optimization based on literature reviews. The samples were named POL-0%, POL-2%, POL-4%, POL-6%, and POL-8% according to the dosage of the POL in the asphalt.

### 2.3. Experimental Methods

#### 2.3.1. Physical Performance Tests

The penetration, softening point, and ductility indexes of different asphalt samples were determined using JTG 3410-2025 [23], and the results of the above tests were utilized to examine the impact of POL on the physical properties of the asphalt binder matrix.

#### 2.3.2. Separation Test

**Figure 2:**
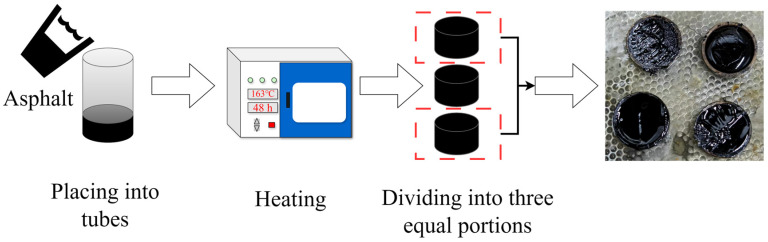
Process of separation test of polyolefin-modified asphalt.

The storage stability of the POL-modified asphalt was evaluated according to the segregation test specified. The pretreated asphalt samples were poured into separation tubes and maintained at 163 ± 1 °C for 48 h, then frozen at 12 °C for 4 h [28]. The tubes were kept sealed throughout the test to prevent external interference. After storage, samples were collected from both the top and bottom sections of each tube, and the difference in softening point between the two layers was determined. Each test was conducted three times, and the average value of the softening-point difference was recorded as the final result. The storage stability of the modified asphalt was assessed based on this difference: a value of ≤2 °C indicates good stability, while a higher value reflects noticeable segregation during storage. The testing procedure is illustrated in Figure 2.

#### 2.3.3. Thermogravimetric Analysis

By evaluating mass variations and thermal transitions over a range of temperatures, the influence of POL on the thermal stability of asphalt was comprehensively assessed. The operational procedure for the simultaneous thermal analysis was as follows: the molten asphalt sample was uniformly spread on the bottom of the crucible, and its mass was recorded. The heating rate was set to 10 °C/min, and the testing temperature was controlled within 50–600 °C. The cooling water circulation system was activated, and nitrogen was introduced at a flow rate of 40 mL/min to maintain a stable inert atmosphere. The thermal analysis test was then initiated, during which the instrument continuously recorded changes in sample mass and heat flow.

#### 2.3.4. Rotational Viscosity Test

The rotational viscosity of the asphalt binder was measured using the Brookfield viscosity method. Preheated asphalt was loaded into the sample cylinder, and the rotor was maintained at the experimental temperature for 1.5 h. The rotor speed was set to 20 rpm, with torque ranging from 10% to 98%. Each sample was tested in triplicate. At each temperature, measurements were recorded at 60 s intervals, with three consecutive readings averaged to obtain the final value. The effect of POL on the workability of asphalt was evaluated using viscosity–temperature curves.

#### 2.3.5. Dynamic Shear Rheometer (DSR) Test

A DSR was utilized in this research to assess the impact of POL on the rheology of asphalt. For testing, the asphalt sample was placed between 25 mm DSR plates, the temperature sensor was inserted into the asphalt, and the gap between the upper and lower plates was adjusted to 1 mm. The experimental process was divided into a high-temperature section (30–80 °C), a medium-temperature section (15–30 °C), and a low-temperature section (−10–30 °C) for temperature scanning. In stress control mode, the composite modulus, phase angle, energy storage modulus, and loss modulus of the asphalt were determined at a constant rate of 10 rad/s [6].

#### 2.3.6. Fourier-Transform Infrared Spectrum (FT-IR) Test

FT-IR analysis can be utilized to explore the impact of POL on the functional group structure of asphalt samples. The 0.1 g sample was dissolved in 2 mL CS_2_ to obtain a solution of 5 wt.%. The solution was dropped in the groove of the potassium bromide wafer until the CS_2_ was fully evaporated. The equipment model was the Nicolet™ iS50 FTIR Spectrometer, manufactured by Thermo Fisher Scientific (Shanghai, China). The resolution and scan times during the test was 4 cm^−1^ and 32 times, and the wave number was 500–4000 cm^−1^.

#### 2.3.7. Fluorescence Microscopy (FM) Test

The FM experiment was used in this research to observe the distribution state of POL in the asphalt. The asphalt sample was prepared using the hot-pressing method: the asphalt sample, glass slide, and cover slip were heated at 160 °C until the asphalt achieved sufficient fluidity. Subsequently, 1–2 drops of asphalt sample were added to the slide and compacted with a cover slip to prepare the asphalt sample slide and moved to the microscope and adjusted until the distribution of POL in the asphalt sample could be clearly observed. FM was employed primarily as a qualitative visualization tool to illustrate the spatial distribution and relative dispersion of the polyolefin phase within the asphalt.

## 3. Results and Discussion

### 3.1. Penetration, Softening Point, and Ductility

**Figure 3:**
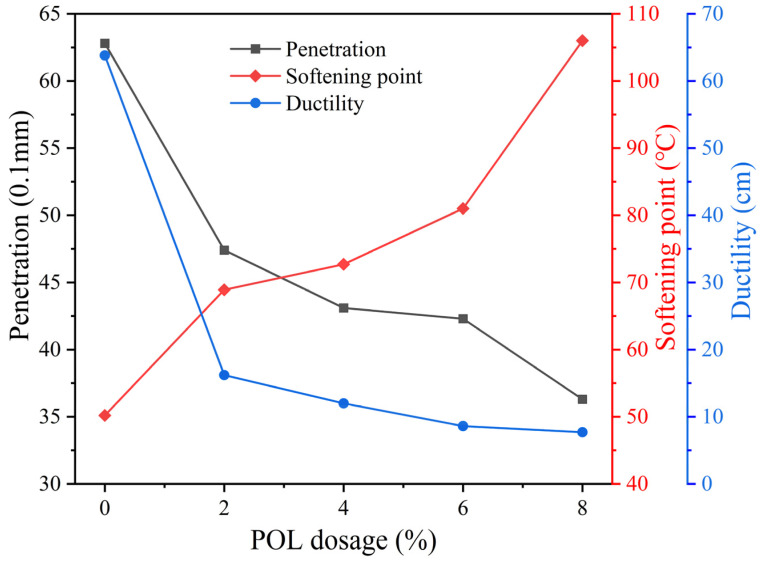
Physical indexes of asphalt with different polyolefin dosage.

Penetration is a key indicator of asphalt consistency, where a lower value reflects higher stiffness and improved resistance to high-temperature deformation [29,30]. The softening point represents asphalt’s temperature sensitivity, with higher values indicating better high-temperature performance [31,32]. As shown in Figure 3, the penetration decreases and the softening point increases with increasing POL dosage. When the dosage rises from 0% to 6%, penetration decreases by 26% while the softening point increases by 35%, suggesting enhanced thermal stability. Further increasing the POL dosage to 8% continues to improve the asphalt performance; however, the incremental enhancement becomes less pronounced compared with that observed at lower dosages.

Ductility reflects the plasticity and toughness of asphalt, where lower values denote higher brittleness and poorer low-temperature performance [33]. Figure 3 shows that ductility sharply decreases with increasing POL dosage due to the enlarged intermolecular spacing induced by POL molecular chains. The ductility of asphalt with 2% POL decreases by about 40% compared with the base asphalt, while further increases to 4% and 6% cause gradual reductions of 6.25% and 6.13%, respectively. Beyond 6%, the ductility remains nearly unchanged. POL addition enhances the high-temperature deformation resistance of asphalt but reduces its low-temperature flexibility. Considering the combined effects of penetration, softening point, and ductility, a 6% POL dosage by asphalt mass is recommended to achieve balanced high- and low-temperature performance.

### 3.2. Rotational Viscosity

**Figure 4:**
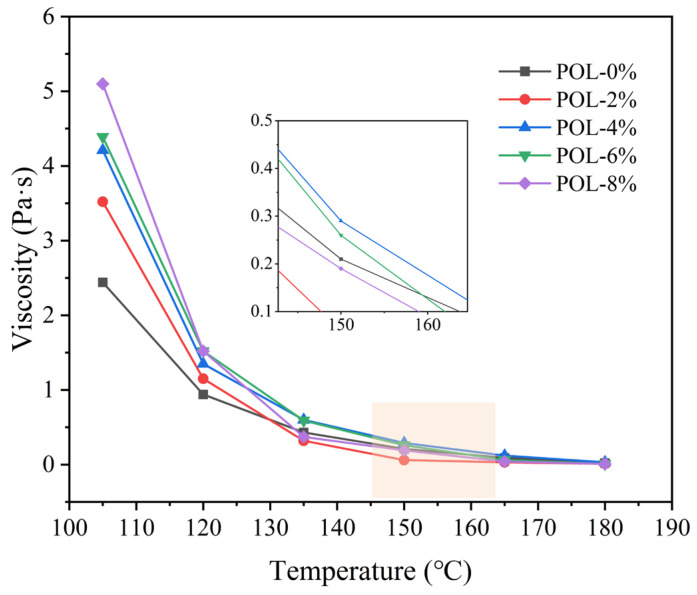
Viscosity–temperature curve of asphalt with different polyolefin dosage.

Rotational viscosity reflects the flow resistance and construction workability of asphalt. A lower viscosity at high temperatures indicates better workability and reduced susceptibility to flow deformation [34,35]. As shown in Figure 4, the addition of POL increases the rotational viscosity of asphalt within 105–130 °C, indicating reduced fluidity. At 105 °C, the viscosity of asphalt containing 2%, 4%, 6%, and 8% POL is 1.44, 2.92, 3.05, and 3.54 times higher, respectively, than that of the base asphalt. This increase results from the adsorption of light components by POL and the swelling of POL molecules in asphalt, which enhances consistency and improves resistance to rutting deformation under high temperatures.

When the temperature exceeds the melting point of POL, the modifier softens and flows, increasing the internal fluidity of asphalt and thus reducing viscosity. Above 135 °C, all samples exhibit low viscosity values similar to the base asphalt. According to the viscosity–temperature relationship, viscosities of 0.17 ± 0.02 Pa·s and 0.28 ± 0.03 Pa·s correspond to the optimal mixing and compaction temperatures, respectively [36]. The mixing and compaction temperatures of asphalt containing 2% POL are approximately 3–5 °C lower than those of the base asphalt, while those of 4–8% POL mixtures remain almost unchanged, indicating that POL modification does not adversely affect the workability of asphalt during construction.

### 3.3. Storage Stability

**Figure 5:**
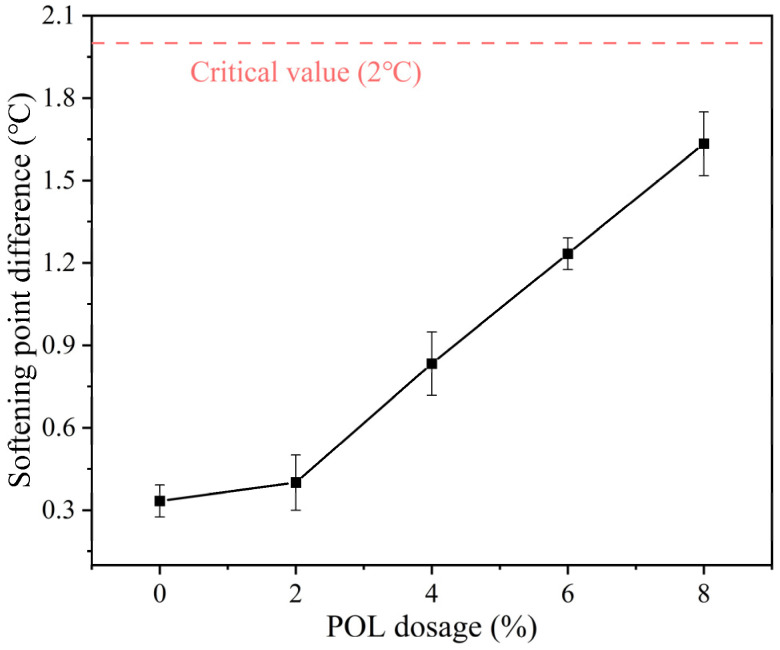
Softening point difference of asphalt with different polyolefin dosages.

The softening point difference obtained through the segregation test can be used to quantitatively evaluate the storage stability of asphalt [37,38,39]. A smaller softening point difference indicates better storage stability. Figure 5 presents the segregation test results of asphalt samples with varying POL dosage and the error bars represent the standard deviation obtained from three repeated separation tests.

As shown in Figure 5, when the POL dosage is 2%, 4%, 6%, and 8%, the softening point differences in the asphalt samples are 0.3 °C, 0.4 °C, 0.8 °C, 1.4 °C, and 1.6 °C, respectively. This indicates that under high-speed shearing and elevated temperatures, the POL can effectively melt and integrate into the asphalt, forming a stable system. Although the softening point difference gradually increases with higher POL dosage, all values remain below 1.5 °C, showing only a slight variation. This meets the specification requirement of a softening point difference not exceeding 2 °C and satisfies the criteria for storage stability. These results demonstrate that POL and asphalt exhibit good storage stability and compatibility. Across different dosage levels, the blend maintains favorable physical properties and can meet the demands of long-term storage.

### 3.4. Thermal Stability

**Figure 6:**
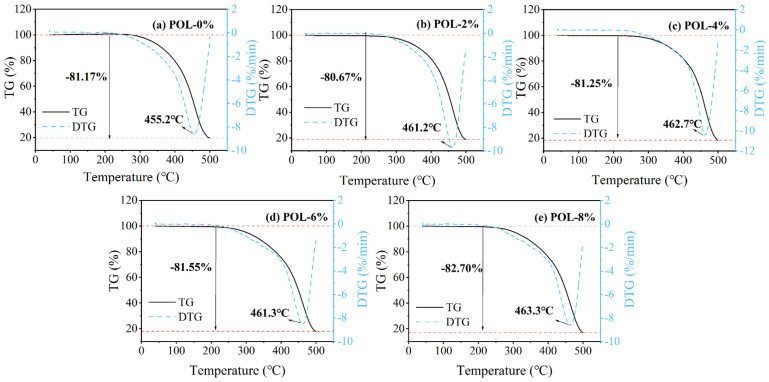
TG-DTG curves of asphalt with different polyolefin dosages.

Figure 6 presents the TG and DTG curves of asphalt binders with different POL dosages. All samples exhibit a distinct weight loss stage within the range of 100–500 °C, which corresponds to the thermal decomposition of light fractions and macromolecular structures in the asphalt. The incorporation of POL consistently shifts the DTG peak toward higher temperatures, increasing the maximum decomposition temperature from 455.2 °C for the unmodified binder to approximately 461–463 °C for the POL-modified asphalt. This upward shift indicates that POL enhances the thermal stability of the asphalt and delays the onset of its principal degradation reactions. In contrast, the total mass loss remains within 80–83% regardless of POL dosage, and a slight increase in mass loss is observed at POL-8%. This behavior suggests that, although POL reinforces the thermal resistance of the asphalt network, the decomposition of POL itself contributes additional volatile products that partially offset the stability improvement. The widening and mild right-shift of the DTG peaks further indicate a more distributed degradation process resulting from the coexistence of multiple thermally active components.

### 3.5. High-Temperature Rheological Properties

#### 3.5.1. Composite Modulus (G*) and Phase Angle (*δ*)

**Figure 7:**
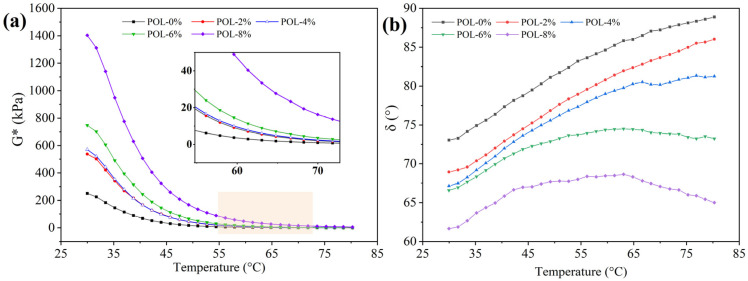
G* and *δ* of asphalt with different polyolefin dosages in high-temperature. (**a**) G*; (**b**) *δ*.

The stiffness of asphalt is characterized by the composite modulus G*. A higher G* value indicates greater rigidity and improved resistance to deformation at high temperatures [40]. As shown in Figure 7a, the G* of all asphalt binders decreases with increasing temperature, consistent with the softening of asphalt in hot conditions that can lead to rutting under load. At the same temperature, the G* of asphalt increases with POL dosage, suggesting that the polymer carbon chains act as a high-modulus reinforcement within the asphalt matrix and enhance its elastic response to external stress.

The viscoelastic behavior of asphalt is represented by the phase angle *δ*. A smaller *δ* value corresponds to a higher elastic component, enabling the asphalt to resist permanent deformation more effectively [41]. As shown in Figure 7b, the *δ* of asphalt containing 2% and 4% POL increases with temperature, indicating that lower dosages do not significantly improve the material’s deformation resistance. When the POL dosage reaches 6% or 8%, *δ* first increases and then decreases with temperature. For asphalt with 6% POL, a *δ* within 70–80 °C is comparable to that of the base asphalt within 50–60 °C, suggesting restricted molecular chain movement and reduced viscous response. At 8% POL, *δ* at 70–80 °C approaches that of the base asphalt at 35–40 °C, reflecting a dominant elastic behavior. These results indicate that increasing POL dosage enhances the high-temperature rheological performance of asphalt by improving its elasticity and deformation resistance.

#### 3.5.2. Rutting Factor (G*/*sinδ*)

**Figure 8:**
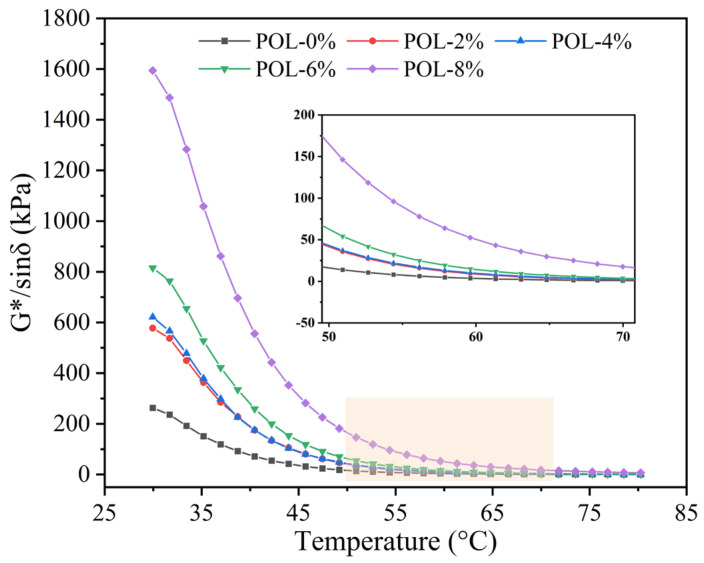
G*/*sinδ* of asphalt with different polyolefin dosages.

G*/*sinδ* can assess asphalt’s ability to resist rutting deformation, with higher values indicating greater rutting resistance [42]. Figure 8 describes the variation in G*/*sinδ* for asphalt with different POL dosage. As the temperature rises, the G*/*sinδ* value of all asphalts decreases, which indicates that asphalt is gradually changing to viscous state, confirming that road surfaces are prone to rutting damage in the high temperatures of summer. Moreover, increasing of POL dosage, G*/*sinδ* value of samples increases to 2–8 times that of asphalt. Therefore, POL has the effect of enabling asphalt to resist rutting disasters at high temperatures. Overall, to increase the ability of asphalt to resist deformation, the POL dosage should be as high as possible.

### 3.6. Fatigue Properties

**Figure 9:**
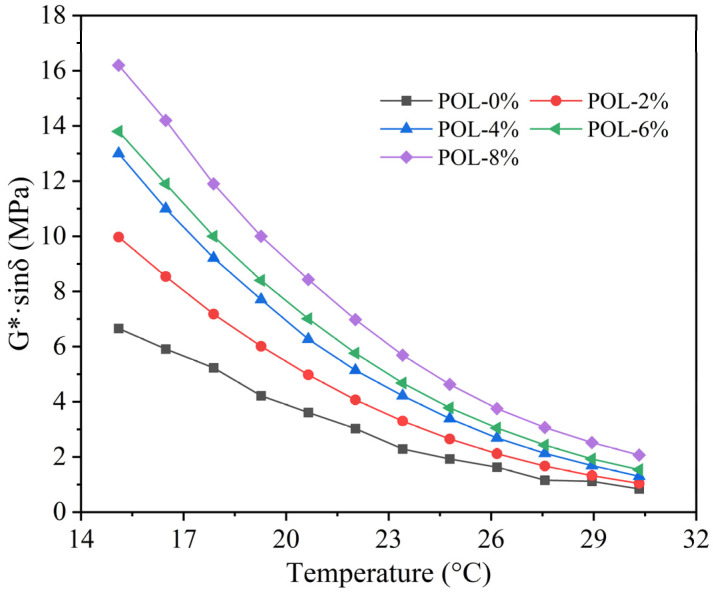
G*·*sinδ* of asphalt with different polyolefin dosages.

The fatigue factor G*·*sinδ* is commonly used to evaluate the cracking resistance of asphalt binders. A lower value of G*·*sinδ* at intermediate temperatures indicates higher material flexibility and an enhanced resistance to fatigue cracking [43]. As shown in Figure 9, the G*·*sinδ* of asphalt decreases with increasing temperature. Higher temperatures facilitate the movement of molecular chain segments, reducing stress concentration and thus improving fatigue resistance.

However, the incorporation of POL increases the G*·*sinδ* value of asphalt, indicating a decline in its fatigue resistance. The higher the POL dosage, the greater the G*·*sinδ* at the same temperature, suggesting that excessive stiffness from the modifier weakens the binder’s ability to dissipate stress. Under repeated loading, POL-modified asphalt tends to experience more pronounced deformation and crack propagation, resulting in reduced fatigue durability. Considering the combined effects of high-temperature rheological performance, thermal stability, and fatigue resistance, a POL dosage of 6 wt.% is recommended as the optimal dosage to achieve a balance between stiffness enhancement and flexibility.

### 3.7. Low-Temperature Rheological Properties

#### 3.7.1. G* and *δ*

**Figure 10:**
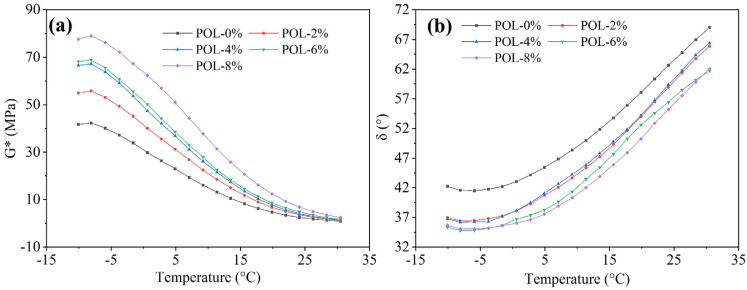
G* and *δ* of asphalt with different polyolefin dosages in low-temperature. (**a**) G*; (**b**) *δ*.

At low temperatures, a relatively lower G* indicates reduced binder stiffness, which facilitates stress relaxation and consequently decreases the susceptibility of asphalt to low-temperature cracking. Figure 10 shows the G* and *δ* of asphalt with five different POL dosages at low temperatures. The G* of the samples increases with the increasing POL dosage, which shows that POL increases the brittleness of asphalt at low temperatures and reduces the low-temperature performance of asphalt. The small *δ* value of the asphalt at low temperatures indicates the insufficient low-temperature crack resistance of the asphalt. As the temperature rises, the proportion of viscous components in the asphalt increases while the proportion of elastic components decreases, resulting in the *δ* values of the five types of asphalt gradually increasing with the increase in temperature. However, at the same temperature, the *δ* of the asphalt decreases with increasing POL dosage, indicating that POL decreases the low-temperature performance of asphalt, which is consistent with the results of the asphalt ductility experiment.

#### 3.7.2. Storage Modulus (*G*′) and Loss Modulus (*G*″)

**Figure 11:**
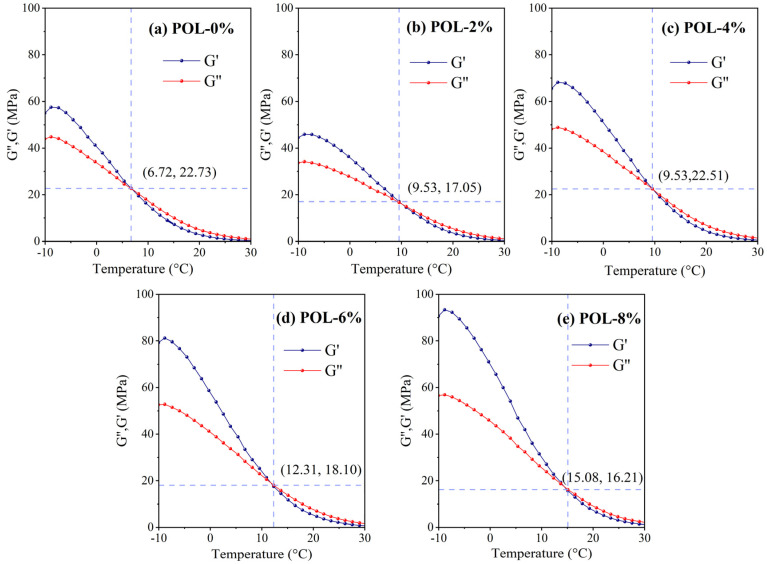
*G*′ and *G*″ of asphalt with different polyolefin dosages.

The storage modulus *G*′ reflects the ability of asphalt to recover its original shape after load removal, while the loss modulus *G*″ represents the energy dissipation associated with viscous deformation and thus reflects asphalt’s viscosity [44]. Figure 11 shows the *G*′ and *G*″ of the asphalt samples with varying POL dosages. Both *G*′ and *G*″ decrease with increasing temperature; however, the decrease in *G*′ is more pronounced, indicating that the viscous component of asphalt becomes dominant at higher temperatures.

In the temperature range of −10 to 30 °C, the *G*′ and *G*″ curves intersect. The intersection temperature characterizes the viscoelastic balance of asphalt: below this temperature, *G*′ > *G*″ indicating elasticity dominates, whereas above it, *G*″ > *G*′ indicating viscous behavior dominates. A lower intersection temperature corresponds to stronger low-temperature viscoelasticity and better low-temperature performance. Figure 11 shows that the intersection temperature increases with POL dosage, suggesting that POL negatively affects low-temperature rheology. Compared with the base asphalt, 2% POL raises the intersection temperature by 41%. Increasing POL from 2% to 4% results in a smaller additional rise of 6.3%, whereas a further increase from 4% to 6% elevates it by 15.7%. When the POL dosage reaches 8%, the intersection temperature rises sharply by 22%, corresponding to a 111.8% increase relative to the base asphalt, indicating severe low-temperature performance deterioration. To mitigate the adverse effect of POL on low-temperature rheology while maintaining high-temperature performance, an optimal POL dosage of 6% by asphalt mass is recommended.

### 3.8. FT-IR Analysis

**Figure 12:**
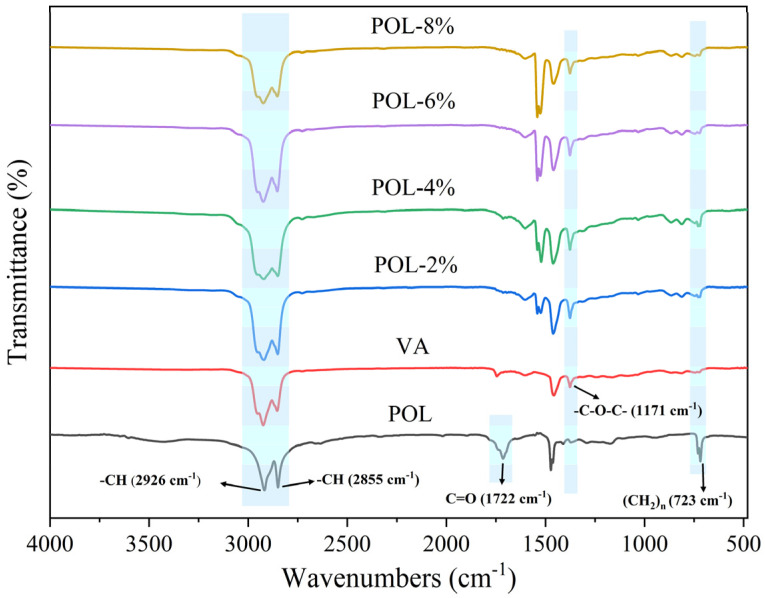
FT-IR test results of asphalt with different polyolefin dosages.

Figure 12 presents the FT-IR spectra of VA, POL, and POL-modified asphalts with varying dosages. No new characteristic absorption peaks appear in the spectra of POL-modified asphalts, indicating that the modification primarily occurs through physical blending rather than chemical reactions between POL and asphalt. A slight increase in the intensity of the -CH stretching bands at 2926 and 2855 cm^−1^ is observed with increasing POL dosage, reflecting the introduction of long-chain aliphatic structures from POL. This suggests that POL reinforces the hydrocarbon skeleton of the binder, enhancing molecular packing and structural stability.

The relative intensity of the carbonyl peak at 1722 cm^−1^ remains essentially unchanged with POL incorporation, indicating minimal influence on the oxidation susceptibility of asphalt at the molecular level during preparation. This observation aligns with the TG results, which show that improved thermal stability originates from delayed decomposition rather than suppressed oxidation.

### 3.9. FM Analysis

**Figure 13:**
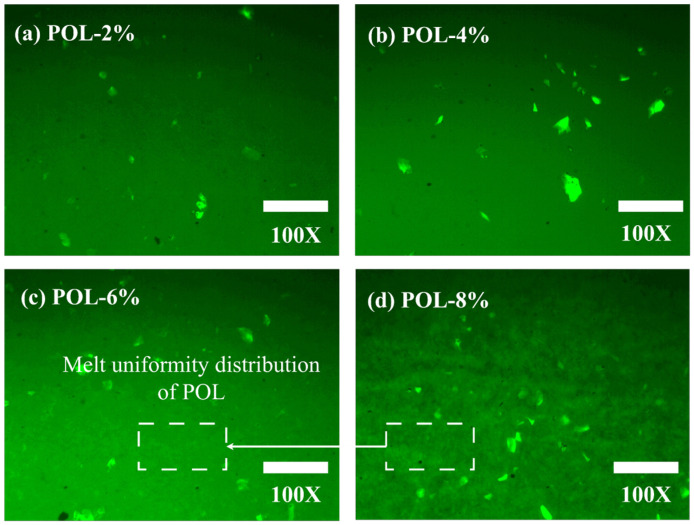
FM results of asphalt with different polyolefin dosage.

Figure 13 illustrates the microscopic distribution of POL in asphalt at different dosages. At 2% POL, distinct agglomerates are observed, indicating limited dispersion and weak interfacial interaction with the asphalt binder. Increasing the dosage to 4% produces finer POL particles with a more uniform distribution, although localized aggregation is still detectable.

At 6% POL, the modifier exhibits the most uniform melting and compatibility within the asphalt, suggesting effective interaction with the colloidal structure and the formation of a stable composite network. At 8% POL, while the overall distribution remains relatively uniform, partial and incomplete melting and phase separation appear. This behavior may result from increased melt viscosity at higher POL dosages, which restricts dispersion and reduces modification efficiency. These microscopic distribution patterns correspond closely with macroscopic performance. Appropriate POL dispersion enhances the structural framework of asphalt, promotes molecular packing and colloidal stability, and contributes to improved high-temperature rutting resistance.

## 4. Conclusions

In this research, the influence of POL dosage on the physical, storage, rheological, and micro-mechanisms of virgin asphalt is systematically quantified. The principal findings are summarized below.

(1) Storage stability tests indicate that the softening-point difference of all POL-modified asphalt remains below 1.5 °C as the POL dosage increases, fully meeting the regulatory threshold of 2 °C. These results confirm the excellent compatibility and storage stability of POL within the asphalt matrix.
(2) Thermogravimetric analyses show that the incorporation of POL markedly retards thermal decomposition and enhances thermal stability. Across all dosages, the total mass loss of the samples stays within a narrow range of 80–83%. The most pronounced retardation effect occurs at POL loadings of 4–8%, where thermal stability is maximized.
(3) DSR tests demonstrate that POL increases the composite modulus of asphalt, functioning as a high-modulus modifier and improving high-temperature rutting resistance. However, higher POL dosages reduce fatigue resistance and low-temperature rheological performance. Considering the overall performance, a POL dosage of 6% by asphalt mass is recommended.
(4) FM tests reveal that with increasing dosage, POL gradually intertwines into a continuous network within the asphalt. When the dosage reaches 8%, however, partial and incomplete melting or phase separation appears.

The research provides an insight into the physical, rheological, and microstructural characteristics of asphalt modified by low-molecular-weight polyolefin. While the results demonstrate the potential of POL as an effective asphalt modifier, a more systematic evaluation, including short-term and long-term aging, mixture performance, and field-relevant conditions, is required and will be the focus of future research.

## Figures and Tables

**Table 1:** Basic physical parameters of virgin asphalt.

Performance Index	Unit	Result	Requirement	Specification
Penetration/25 °C, 0.1 mm	mm	70.8	60–80	JTG 3410-2025 [23]
Softening point	°C	45.8	≥45	JTG 3410-2025 [23]
Ductility/10 °C, 5 cm/min	cm	63.4	Measured value	JTG 3410-2025 [23]
Boulevard viscosity/135 °C	cp	430.0	Measured value	JTG 3410-2025 [23]
Density	g/cm^3^	1.034	Measured value	JTG 3410-2025 [23]

**Table 2:** Performance indicators of polyolefin in this research.

Performance Index	Unit	Indicator Value	Specification
Melting drop point	°C	136	SH/T 0800-2007 [24]
Viscosity/150 °C	cps	4500	SH/T 0739-2003 [25]
Molecular weight	Dalton	2000–5000	GB/T 27843 [26]
Density	g/cm^3^	0.97	ASTMD-1505 [27]

## Data Availability

The original contributions presented in this study are included in the article. Further inquiries can be directed to the corresponding authors.
